# A Chicken-and-Egg Predicament: Malignant Hypertension Versus HIV-Induced Thrombotic Microangiopathic Anemia Conundrum

**DOI:** 10.7759/cureus.64304

**Published:** 2024-07-11

**Authors:** Daniel Zamanian, Akshat Agrawal, Arpita Pawa, Hector J Brunet-Rodriguez, Sreedhara Alla

**Affiliations:** 1 Internal Medicine, Willis Knighton Healthcare System, Shreveport, USA; 2 Internal Medicine, Pt. Jawahar Lal Nehru Memorial Medical College, Raipur, IND; 3 Infectious Disease, Willis Knighton Healthcare System, Shreveport, USA; 4 Nephrology, Willis Knighton Healthcare System, Shreveport, USA

**Keywords:** endothelial damage, end-stage renal failure, severe hypertension, human immunodeficiency virus (hiv)-positive, thrombotic microangiopathy (tma)

## Abstract

Thrombotic microangiopathy (TMA) is a condition characterized by hemolytic anemia, thrombocytopenia, and organ damage due to the formation of microthrombi. It can be classified as primary or secondary, with secondary TMA being associated with conditions such as infections, autoimmune diseases, and malignancies. This report details the case of a 39-year-old male with secondary TMA, exploring the potential roles of malignant hypertension and HIV infection with the aim of examining the potential link between malignant hypertension and HIV infection in the development of TMA, highlighting the need for a thorough and broad diagnostic approach.

## Introduction

Thrombotic microangiopathy (TMA) involves microangiopathic hemolytic anemia, thrombocytopenia, and organ damage due to microthrombi formation. Primary TMA occurs as a result of thrombotic thrombocytopenic purpura (TTP) and hemolytic uremic syndrome, whereas secondary TMA can result from various conditions such as pregnancy, autoimmune diseases, infections, malignancy, and transplantation, all leading to vascular endothelial damage and microthrombi formation [1,2]. This report presents the case of a 39-year-old male with TMA due to secondary causes, who presented with microangiopathic hemolytic anemia with schistocytes, thrombocytopenia, and acute renal failure.

## Case presentation

**Figure 1:**
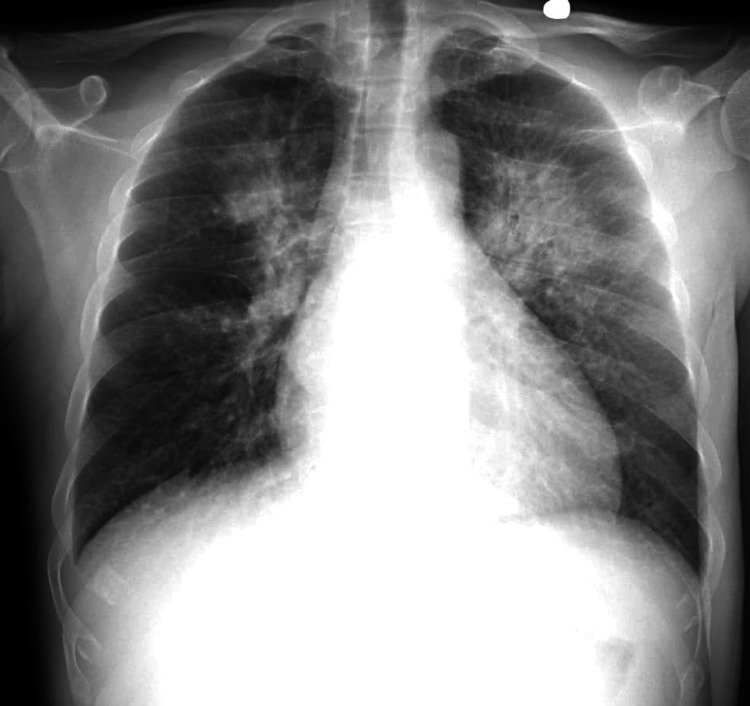
Chest X-ray showing diffuse bilateral infiltrates.

**Figure 2:**
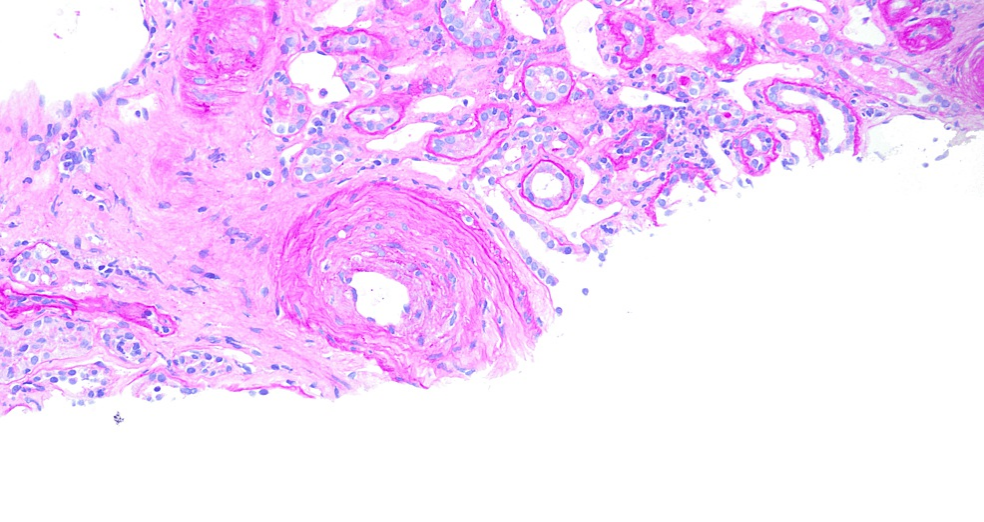
Cortex with PAS stain High-power view (40x) showing small artery with endothelial swelling and mixed interstitial infiltrate.

**Figure 3:**
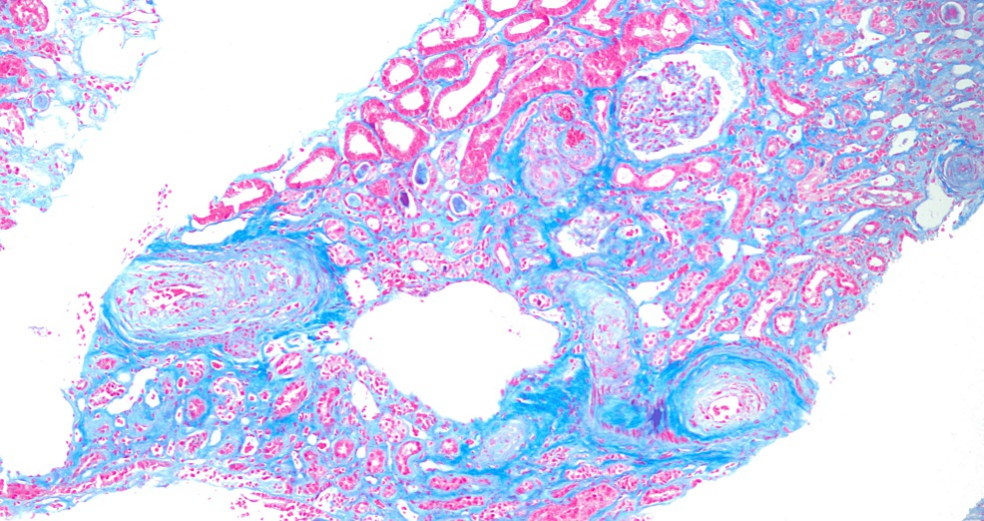
Cortex with trichome stain showing interstitial fibrosis Low-power view (10x) highlighting the prominent fibrin thrombus involving the arteriole.

**Figure 4:**
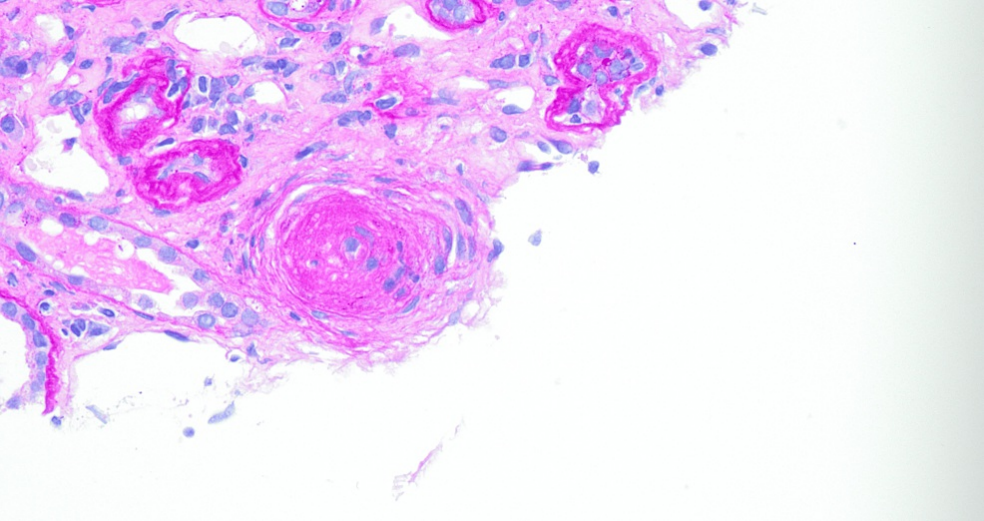
Arteriole showing myointimal thickening High-power view (40x) showing thrombosed arteriole with fibrinoid necrosis of the vessel wall.

**Figure 5:**
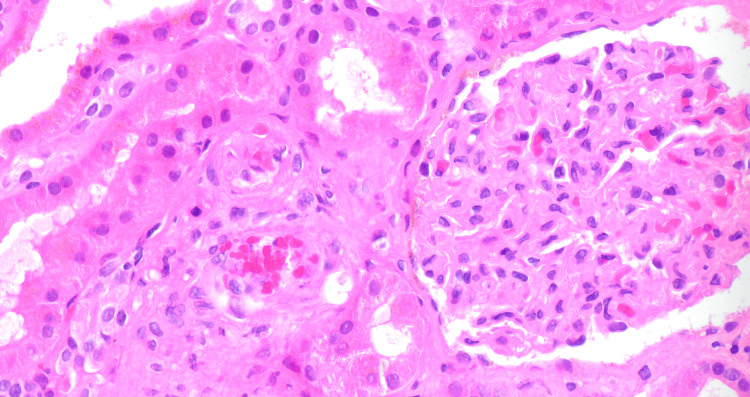
Schistocytes within the vessel High-power view (40x) showing glomerulus with endothelial swelling giving it a bloodless appearance and collapse of tuft along with entrapment of red blood cells.

**Figure 6:**
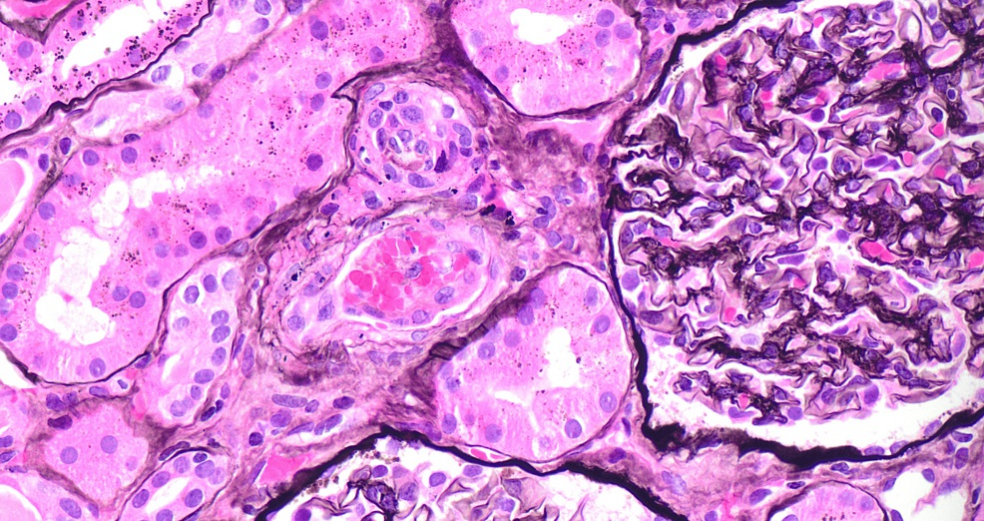
Thrombotic microangiopathy in the vessel High-power view (40x; Jones methenamine silver stain) showing argyrophilic nature of fibrin within the arteriole and staining of the basement membrane of glomeruli.

A 39-year-old male presented to the hospital with symptoms of sore throat, weakness, dizziness, and rash on bilateral lower extremities over four days. He had a history of hypertension, marijuana abuse, and a traumatic neck injury. On admission, vitals were significant for blood pressure of 179/122 mm Hg, heart rate of 103 beats/min, respiratory rate of 15 breaths/min, and oxygen saturation of 93%, and he was afebrile. Physical examination revealed lower extremity erythema and +1 bilateral lower extremity edema. laboratory findings showed mild anemia (hemoglobin of 7.2 g/dL), thrombocytopenia (platelet count of 70 x 10^9^/L), anion gap metabolic acidosis (HCO_3_ 12 mmol/L, anion gap 22 mmol/L), and elevated creatinine (13.9 mg/dL). Chest X-ray revealed diffuse bilateral infiltrates concerning for volume overload versus pneumonia (Figure 1). The patient had decreased urine output, with urinalysis showing +3 urine protein, +3 urine occult blood, and a urine protein creatinine ratio of 2.7 mg/g. Serology was positive for HIV-1 and HIV-2 antigen-antibody tests, with a viral load of 135,000 copies/mL and a CD4 count of 45 cells/mm3. Opportunistic infection workup was positive for *Pneumocystis jirovecii*, and appropriate treatment was initiated. The patient was not started on highly active antiretroviral therapy (HAART) at the time due to consideration for immune reconstitution inflammatory syndrome. The patient’s hematological parameters prompted a bone marrow biopsy, which was reported as normal. Glucose-6-phosphate dehydrogenase levels, vitamin B12, and folate and copper levels were normal, and direct antiglobulin test (DAT) was also negative. The patient’s last available medical records did not show that he was on any anti-hypertensive medications, and laboratory findings that could point to secondary causes of hypertension were within normal limits. The patient was assumed to have essential hypertension and treated as such given the urgency of the presentation. The patient continued to be oliguric with worsening renal function and was ultimately initiated on hemodialysis. ADAMTS13 (a disintegrin and metalloprotease with thrombospondin type motifs) levels, serum complement levels, and a kidney biopsy were ordered at this point to rule out TTP and atypical hemolytic syndrome (aHUS). ADAMTS13 levels were 40%, and the serum C3 was slightly low at 78 mg/dL with normal C4 levels. The kidney biopsy revealed tubular atrophy with minimal mixed interstitial infiltrate (Figure 2) and severe interstitial fibrosis (Figure 3). Arterioles showed severe myointimal thickening with endothelial swelling, intimal edema, and fragmented RBC (Figure 4), with the presence of schistocytes (Figure 5). The biopsy suggested TMA in the kidney (Figure 6), with chronic hypertensive changes. Immunofluorescence evaluation of the biopsy sample was negative for IgA, IgG, IgM, C3, and light chains, with electron microscopy showing no immune deposits in the glomerular basement membrane and mesangium. The laboratory and pathology findings suggested against TTP and aHUS. The patient continued to receive hemodialysis with blood pressure control, and his laboratory parameters showed improvement with improving kidney function and decreasing lactate dehydrogenase. HLA B5071 assay was also performed, which was negative. He was discharged with antihypertensive medication with instructions for continued outpatient hemodialysis due to the development of end-stage renal disease, as well as continued treatment for *Pneumocystis jirovecii* pneumonia with outpatient infectious disease follow-up. He was also initiated on HAART after discharge.

## Discussion

On admission, the patient had malignant hypertension, microangiopathic hemolytic anemia, thrombocytopenia, and acute renal failure, along with high HIV viral loads and low CD4 counts. Similar presentations have been noted in cases of active HIV with low CD4 counts and poor HAART compliance [3]. The mechanism of HIV-induced TMA is unclear, with one possible mechanism involving von Willebrand factor release due to direct endothelial injury by HIV viral proteins, leading to platelet aggregation and microthrombi formation [4].

In addition, malignant hypertension triggers endothelial injury via sheer stress, activating RAAS (renin-angiotensin-aldosterone system) and causing release of angiotensin II, which directly causes damage to vessels resulting in endothelial dysfunction [5]. This case highlights the interplay of malignant hypertension and active HIV causing underlying TMA leading to acute renal failure. There could have been several scenarios with regard to the TMA in this patient; one is that HIV infection triggered TMA and as a result caused acute renal failure. Then the acute renal failure worsened pre-existing hypertension and resulted in hypertensive crisis. Another scenario could be that malignant hypertension caused TMA, which resulted in acute renal failure, and the patient was also newly diagnosed with HIV during hospitalization. There is also the possibility of interplay between malignant hypertension and HIV, with both contributing to TMA, or it could be that the patient might have had pre-existing HIV infection, which caused a chronic state of inflammation. Chronic state of inflammation contributed to malignant hypertension (secondary to endothelial dysfunction), and malignant hypertension eventually triggered acute renal failure. Determining the primary cause is challenging given the patient's history and biopsy result.

## Conclusions

This case highlights a possible interplay between HIV and malignant hypertension as the cause of TMA, which ultimately led to the development of acute renal failure in a 39-year-old patient. Although malignant hypertension has been known to cause TMA, HIV might be as a lesser known cause for it. What makes this case interesting is the possible pathophysiology of why this happened. The mechanisms by which HIV can cause TMA are varied, leading to renal failure. Renal failure itself can worsen an already pre-existing hypertension due to the kidneys' impaired ability to regulate blood pressure through fluid balance and hormone release. Renal failure exacerbates hypertension through increased sodium and water retention, activation of RAAS, reduced nitric oxide availability impairing blood vessel dilation, and hormonal imbalances such as elevated aldosterone levels, all contributing to elevated blood pressure. Alternatively, it is possible that the HIV infection is incidental and not related to the TMA and that the TMA was primarily caused by malignant hypertension. Understanding this will not only shed light on knowing about complications of HIV but also enable physicians to have a more in-depth look and broader differential when it comes to diagnosing and managing a patient with this condition. Although the biopsy indicated a hypertensive process, the presence of HIV infection underscores the need to consider overlapping etiologies. This underscores the importance of a broad approach to diagnosing TMA.
